# Efficacy of Pain Neuroscience Education Combined with Exercise in Older Adults with Chronic Pain: Study Protocol for a Randomized Controlled Trial

**DOI:** 10.3390/jcm15103696

**Published:** 2026-05-11

**Authors:** Javier Torres-Alonso, Luis Polo-Ferrero, Sara Hernández-Rubia, María Carmen Sánchez-Sánchez, Ana Silvia Puente-González, Susana Sáez-Gutiérrez, Roberto Méndez-Sánchez, Fausto J. Barbero-Iglesias

**Affiliations:** 1 Department of Nursing and Physiotherapy, Universidad de Salamanca, 37007 Salamanca, Spain; javiertorres@usal.es (J.T.-A.); sarahernandezrubia@usal.es (S.H.-R.); csanchez@usal.es (M.C.S.-S.); silviapugo@usal.es (A.S.P.-G.); susanasg@usal.es (S.S.-G.); fausbar@usal.es (F.J.B.-I.); 2 Institute of Biomedical Research of Salamanca (IBSAL), 37007 Salamanca, Spain

**Keywords:** aged, chronic pain, pain neuroscience education

## Abstract

**Background/Objectives:** Chronic pain in older adults is a highly disabling epidemic, often overtreated with passive pharmacological approaches. Although clinical guidelines recommend active strategies combining multicomponent exercise and Pain Neuroscience Education (PNE), robust geriatric evidence remains scarce. Therefore, the central objective of this randomized controlled trial (RCT) is to evaluate the efficacy of combined PNE and exercise versus exercise alone. Crucially, this RCT addresses a major literature gap by investigating the long-term dose–response effect of PNE (an 8-week intensive vs. a 32-week maintenance program) to determine the optimal strategy for sustaining behavioral change and pain relief. **Methods:** A prospective, single-blind RCT (1:1:1 allocation) will recruit 90 older adults (≥65 years) with primary chronic musculoskeletal pain. The 32-week intervention comprises three arms: a control group (multicomponent exercise), Intervention Group 1 (exercise + 8 weeks of PNE), and Intervention Group 2 (exercise + 32 weeks of PNE). The primary outcome is pain intensity (assessed via the Numeric Rating Scale [NRS]). Secondary outcomes include kinesiophobia, pain catastrophizing, chronic pain grade and related disability, quality of life, physical performance, body composition and analgesic consumption. Data collected at baseline, 8, and 32 weeks will be analyzed using mixed-effects models for repeated measures. **Results:** As this is a study protocol, there are no results to report yet. Upon completion of the trial, data will be analyzed using mixed-effects models for repeated measures to evaluate intra- and intergroup changes over time, and the findings will be disseminated in future publications. **Conclusions:** By evaluating the long-term dose–response effect, this study will determine the optimal PNE dosage required for sustained pain relief and behavioral change in older adults. If our hypotheses are confirmed, the findings will generate high-quality evidence to support the integration of combined active interventions into community settings, promoting active aging and reducing the burden of chronic pain. **Trial Registration:** ClinicalTrials.gov Identifier: NCT07287501.

## 1. Introduction

Population aging constitutes a major public health challenge, with chronic pain standing out as a highly prevalent and disabling condition. Approximately 36.6% of older adults suffer from persistent pain, which negatively impacts their mobility, autonomy, and mood, increasing the risk of frailty, falls, and cognitive decline [1,2,3,4,5,6,7]. Pathophysiologically, chronic pain in this population is a complex phenomenon driven by age-related central sensitization and maladaptive neuroplasticity, where pain loses its protective function [8,9]. Furthermore, psychosocial factors such as kinesiophobia (fear of movement) and pain catastrophizing play a critical role in its chronification, perpetuating a vicious cycle of inactivity, physical deconditioning, and functional disability [10,11,12,13].

Despite its multidimensional nature, the therapeutic approach often remains predominantly biomedical and pharmacological, posing significant risks of polypharmacy and adverse effects in older adults [14,15]. Consequently, current clinical guidelines strongly advocate a paradigm shift toward active, non-pharmacological interventions, highlighting therapeutic exercise and PNE to facilitate active coping [16,17,18,19,20,21,22,23,24].

PNE aims to reconceptualize the pain experience by explaining the underlying neurophysiological mechanisms (such as central sensitization and neuroplasticity), thereby reducing the perception of threat and facilitating an active coping approach [25,26]. Conceptually, PNE is deeply rooted in the Fear-Avoidance Model and cognitive–behavioral frameworks [27]. By explaining the neurobiology of pain, PNE successfully reduces pain catastrophizing and kinesiophobia [28,29]. Recent mediation analyses demonstrate that this reduction in fear of movement is the primary mechanism leading to long-term functional recovery [30]. By disrupting avoidance behaviors, PNE significantly increases patients’ pain self-efficacy (their confidence to move despite the pain) [31]. This enhanced self-efficacy acts as the crucial cognitive–behavioral catalyst that fosters greater long-term adherence to active treatments, such as multicomponent exercise programs [32].

Current evidence strongly supports this combined approach across diverse clinical populations. Recent meta-analyses and trials demonstrate that combining PNE with physical exercise yields significantly greater short- and long-term improvements in pain intensity, disability, and kinesiophobia compared to exercise alone in patients with chronic spinal pain, chronic neck pain, and fibromyalgia [28,33,34,35]. By effectively reducing the perceived threat value of pain and promoting active coping, PNE has established itself as a cornerstone in modern pain management. In the older adult population, emerging trials confirm that adapted PNE programs can effectively improve physical function, step count, and fear-avoidance beliefs [36,37]. However, despite these promising findings, robust empirical evidence adapted to the specific cognitive and physical needs of the geriatric population remains scarce. Previous studies in older adults often present substantial methodological limitations, high heterogeneity, and a lack of long-term follow-ups [38,39,40,41]. Furthermore, beyond these methodological constraints, a major scientific gap remains regarding the optimal dosage of educational interventions required to modify deeply rooted beliefs and sustain behavioral changes over time in geriatrics. A recent dose–response meta-analysis identified that at least 150 to 200 min of PNE are needed to achieve clinically important changes in pain and disability [42], yet long-term comparative studies directly evaluating maintenance protocols in older adults are lacking.

Therefore, this study protocol describes a randomized controlled trial aiming to evaluate the efficacy of a combined program of PNE and multicomponent exercise compared to exercise alone in older adults with chronic pain. Specifically, we will investigate the dose–response effect of PNE by comparing a short-term intensive phase (8 weeks; 240 min) versus a long-term maintenance program (32 weeks; 360 min). The primary objective of this trial is to determine the effectiveness of these interventions in reducing pain intensity, ultimately helping to establish the optimal strategy for sustaining long-term pain relief and promoting active aging.

## 2. Materials and Methods

### 2.1. Study Design

**Figure 1:**
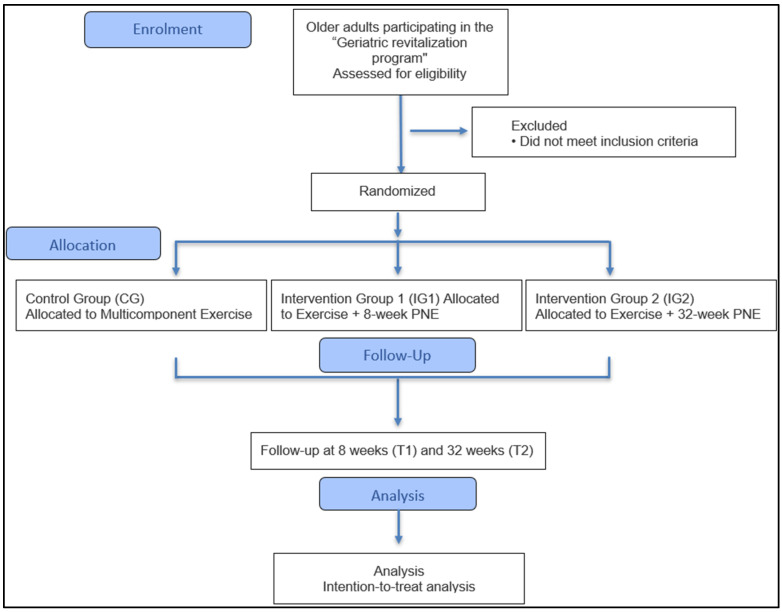
CONSORT flow diagram of the study protocol outlining the enrollment, allocation, follow-up, and planned analysis of participants. CG: Control Group; IG1: Intervention Group 1; IG2: Intervention Group 2; PNE: Pain Neuroscience Education; T1: Evaluation at 8 weeks; T2: Evaluation at 32 weeks.

This study has been designed as a prospective, longitudinal, randomized controlled trial with three parallel groups and a 1:1:1 allocation. The protocol follows the guidelines of the CONSORT (Consolidated Standards of Reporting Trials) statement for non-pharmacological clinical trials [43]. The study will have a total duration of 12 months, with a 32-week intervention period. The flow of participants through each stage of the trial is depicted in Figure 1.

### 2.2. Participants and Eligibility Criteria

The study population will consist of community-dwelling older adults aged 65 years or older (Salamanca, Spain), recruited through the “Programa de Revitalización Geriátrica (PReGe)”. Inclusion criteria are as follows: (1) age ≥65 years; (2) history of primary chronic musculoskeletal pain lasting more than 3 months; (3) pain intensity ≥3 out of 10 on the Numeric Rating Scale (NRS) [44,45]. Exclusion criteria are as follows: (1) presence of secondary chronic pain conditions (e.g., oncological pain, specific rheumatic diseases such as ankylosing spondylitis or rheumatoid arthritis, fibromyalgia, neuralgias, and nerve injuries or neuropathies such as carpal tunnel syndrome); (2) severe psychiatric pathologies; (3) uncontrolled systemic, inflammatory, or cardiac diseases; or (4) other medical conditions that contraindicate physical exercise.

### 2.3. Randomization and Blinding

Following the baseline assessment, eligible participants will be randomly assigned to one of the three study groups using a computer-generated sequence (R software version 4.2.1; R Foundation for Statistical Computing, Vienna, Austria), utilizing a block randomization design to ensure balance between the groups. Allocation will be performed by an independent researcher uninvolved in the recruitment process. Due to the nature of the intervention (exercise and education), it is not possible to blind the participants or the therapists. However, a single-blind design will be employed: the evaluators in charge of recording the outcome variables will be unaware of the group allocation, and the statistical analysis will be performed by an independent researcher using coded data, ensuring they also remain completely blinded to the group distribution and allocation.

### 2.4. Interventions

Interventions will be carried out at university facilities and municipal community centers. All groups will maintain their usual pharmacological treatment and activities of daily living. All interventions will be delivered by physiotherapists who received specific and standardized training in pain neurophysiology and therapeutic education for chronic pain during the pre-clinical phase of the study to ensure treatment homogeneity.

The PNE protocol was meticulously designed based on current high-quality evidence. Specifically, the selection of the thematic contents is grounded in the latest international Delphi consensus on therapeutic pain education [46]. Furthermore, the two-phase pedagogical structure and the use of active constructivist methodologies during the maintenance phase are justified by the educational frameworks recently described by the PETAL collaboration [24] to maximize long-term behavioral change and self-efficacy. Phase 1 (weeks 1–8) focuses on reconceptualizing pain through interactive, age-adapted sessions explaining neurophysiological concepts (e.g., central sensitization, neuroplasticity, damage vs. pain). Phase 2 (weeks 9–32) employs active and constructivist methodologies (such as problem-based learning, clinical case resolution, and role-playing) to consolidate knowledge transfer to daily life activities. Thus, comparing a short-term PNE program with a long-term extended phase is not primarily intended to test a higher cumulative volume of information, but rather the efficacy of sustained engagement and periodic reinforcement in translating the initial reconceptualization of pain into long-term habits and pain self-management. The rationale for selecting these specific durations is twofold. First, the 8-week intensive phase (comprising 16 sessions of 15 min) provides a total educational dose of 240 min. This duration was specifically chosen to exceed the established efficacy threshold, as a recent dose–response meta-analysis concluded that at least 150 to 200 min of PNE are necessary to achieve clinically meaningful improvements in pain and disability [42]. Second, the 32-week duration was selected because it mirrors the standard 8-month academic cycle of municipal health and exercise programs for older adults in the community (such as the PReGe). This allows for a pragmatic evaluation of whether sustaining the educational reinforcement throughout an entire real-world community program cycle yields superior long-term adherence and behavioral changes. The specific characteristics of each arm are detailed in Table 1. The structure, content, and objectives of the multicomponent exercise program are detailed in Table 2, while the PNE session contents and their specific pedagogical objectives are summarized in Table 3.

- Control Group (CG-Multicomponent Training): They will undergo a multicomponent physical exercise program supervised by physiotherapists, based on the recommendations of the LIFE and SPRINTT studies [47,48]. Sessions will include a warm-up, aerobic exercise, muscle strength, balance/coordination and cool-down. Intensity will be individually adjusted using the Borg Rating of Perceived Exertion Scale (target range 5–7) [49]. Because this group receives a structured physical intervention, it functions as an active comparator rather than a traditional passive control. This baseline ensures that any expected between-group differences will specifically reflect the added value of the PNE interventions. The detailed structure, content, and objectives of the exercise sessions are presented in Table 2.
- Intervention Group 1 (IG1-Exercise + PNE 8 weeks): In addition to the same exercise protocol as the CG, participants will receive the Phase 1 PNE program during the first 8 weeks. These 15 min educational sessions will be delivered immediately before the exercise, using visual materials and supporting leaflets to facilitate active coping.
- Intervention Group 2 (IG2-Exercise + PNE 32 weeks): Participants will receive the same exercise program and the initial 8-week Phase 1 PNE program (identical to IG1). Subsequently, they will continue with the Phase 2 educational maintenance program until week 32. This phase consists of biweekly 15 min sessions delivered before the exercise, focusing on group dynamics and useful tools for pain management, such as dietary guidelines and relaxation techniques.

To ensure intervention fidelity and pedagogical standardization across all groups, PNE sessions will be strictly guided by a predefined intervention manual, using standardized visual aids and structured educational leaflets. To actively assess participant comprehension, a dedicated question-and-answer period will conclude each session, and specific knowledge verification questionnaires will be administered during sessions 7 and 14. Furthermore, from session 17 onwards, the implementation of active constructivist methodologies will allow the facilitator to continuously monitor engagement, identify and correct any cognitive misconceptions in real-time, and guide participants toward the accurate practical application of the concepts in their daily lives.

### 2.5. Outcomes and Data Collection

Evaluations will be conducted at three time points: Baseline (T0), Intermediate at 8 weeks (T1), and Final at 32 weeks (T2).

- Primary Outcome: The primary outcome will be pain intensity measured by the NRS [32,33].
- Secondary Outcomes: Psychosocial variables will include Kinesiophobia (Tampa Scale for Kinesiophobia, TSK-11) [50,51], Pain Catastrophizing (Pain Catastrophizing Scale, PCS) [52], chronic pain grade and related disability (Chronic Pain Grade Scale) [53], and health-related quality of life (SF-12) [54,55]. Physical performance will be evaluated using the Short Physical Performance Battery (SPPB) [56], the Timed Up and Go (TUG) test [57], handgrip strength (JAMAR Plus+ dynamometer; Performance Health, Warrenville, IL, USA) [58], and the force–velocity profile of the lower extremities (ADR linear encoder; ADR Inercial, Toledo, Spain) [59]. Changes in body composition (Tanita BC-418 Bioelectrical Impedance; Tanita Corporation, Tokyo, Japan) [60,61] and daily analgesic consumption will also be recorded. Additionally, adherence to the intervention will be rigorously monitored by recording participant attendance at all multicomponent exercise and Pain Neuroscience Education sessions. Finally, the Trail Making Test (TMT) will be administered at baseline to evaluate executive function and cognitive flexibility [62,63]. The inclusion of this instrument is based on an a priori secondary hypothesis that baseline levels of these capacities modulate the responsiveness to PNE, given that this educational intervention requires high cognitive flexibility to successfully reconceptualize the pain experience [22,26].

### 2.6. Sample Size and Statistical Analysis

The sample size was calculated based on the primary outcome (pain intensity via NRS). The estimation was performed using the GRANMO Version 8.0 tool. We calculated the effect size based on a Minimal Clinically Important Difference (MCID) of 1.5 points and a common standard deviation of 1.8. Assuming a two-sided contrast and an Analysis of Variance (ANOVA) for three independent groups, with an alpha level of 0.05, a statistical power of 80%, and an anticipated dropout rate of 10%, a minimum of 30 participants per group is required, resulting in a total sample of 90 subjects.

Intention-to-treat analysis will be performed. The primary endpoint for determining the long-term efficacy of the interventions is defined as the change in the primary outcome (NRS) at 32 weeks. Mixed-effects models for repeated measures will be used to evaluate intra- and intergroup changes over time (T0, T1, T2), considering the Group × Time interaction. To adjust for initial variance, baseline values of each respective outcome will be included as covariates in all models. Within this modeling framework, missing data will be inherently handled using restricted maximum likelihood (REML) estimation under the missing-at-random assumption. Additionally, to control for the inflation of Type I error associated with the analysis of multiple secondary outcomes, the Benjamini–Hochberg False Discovery Rate procedure will be applied. Furthermore, a secondary analysis stratified by sex will be conducted to evaluate potential differences in the intervention’s efficacy between men and women. Additionally, baseline TMT scores will be utilized as a covariate in an a priori secondary analysis to determine if baseline levels of executive function and cognitive flexibility modulate the treatment outcomes. The significance level will be set at *p* < 0.05.

## 3. Ethics and Dissemination

### 3.1. Ethical Approval and Consent to Participate

The study protocol has been reviewed and approved by the Ethics Committee for Research of the University of Salamanca (approval code: 1424). The trial will be conducted in strict accordance with the ethical principles of the Declaration of Helsinki [64]. Prior to enrollment, all eligible participants will receive detailed verbal and written information regarding the study’s objectives, procedures, potential risks, and benefits. Written informed consent will be obtained from all subjects before any study-related procedures are performed. Participation is strictly voluntary, and no financial compensation will be provided. Participants reserve the right to withdraw from the study at any time without any negative consequences for their usual care.

### 3.2. Confidentiality and Data Management

All data collected during the study will be recorded systematically, confidentially, and in an encoded manner. To ensure anonymity, each participant will be assigned a unique alphanumeric identification code. Personal identifying information will not be included in the analysis database. Data protection and privacy will comply with the General Data Protection Regulation (EU) 2016/679 [65] and the Spanish Organic Law 3/2018 on the Protection of Personal Data and Guarantee of Digital Rights [66]. The principal investigator will securely store the correspondence list between the codes and personal identities in a separate, encrypted file, accessible only to authorized research team members.

### 3.3. Dissemination Policy

Upon completion of the study, the global aggregated results will be shared in annual community scientific dissemination events. Additionally, participants will have the option to request a personalized report detailing their individual progress and outcomes regarding mobility, muscle strength, body composition, and pain-related variables throughout the trial. The clinical and scientific findings derived from this protocol will be submitted for publication in peer-reviewed medical journals and presented at national and international scientific conferences.

## 4. Results

As this manuscript represents a study protocol, there are no results to report at this stage. Participant recruitment and data collection are scheduled to be carried out as outlined in the study timeline. Upon completion of the trial, the collected data will be analyzed using the statistical methods described above to evaluate both the intra- and intergroup efficacy of the interventions. The final results will be disseminated through peer-reviewed scientific publications and presentations at national and international conferences.

## 5. Discussion

As populations age, an increasing number of older adults suffer from chronic musculoskeletal pain, a condition that often cannot be effectively managed exclusively through pharmacological treatments or under a traditional biomedical model. There is an urgent need to develop and implement non-pharmacological interventions based on empirical evidence [16,17,18]. To maximize their utility, these interventions must be designed in a way that allows them to be applied by health professionals in different settings (such as primary care centers or community spaces), thereby facilitating older adults’ access to pain management services and promoting self-management in their daily lives.

The present multicomponent intervention has been designed in accordance with these principles, integrating physical training and PNE based on current clinical recommendations. By combining both strategies, this study seeks not only to improve physical functionality but also to equip patients with cognitive tools to modify maladaptive thoughts and behaviors, such as kinesiophobia and catastrophizing, which often perpetuate disability in this population [10,11,12,13]. The protocol will be evaluated through a randomized controlled trial that introduces an innovative approach: comparing the effects of two different doses of education (8 weeks versus a 32-week maintenance phase) combined with exercise, against isolated exercise practice.

Based on this design, our primary hypothesis is that the combined intervention (PNE and exercise) will yield superior improvements in pain intensity, physical functionality, and psychosocial variables (such as kinesiophobia and catastrophizing) compared to exercise alone. Furthermore, we hypothesize that the extended 32-week PNE program will be significantly more effective in sustaining these clinical benefits and behavioral changes over time than the 8-week program. In addition to these clinical outcomes, we introduce an a priori secondary hypothesis regarding the moderating role of baseline cognitive flexibility and executive function, measured via the TMT. Chronic pain, particularly in older adults, often impairs these cognitive capacities [67,68,69]. Concurrently, PNE strictly requires pain reconceptualization, a process of unlearning deeply ingrained biomedical beliefs to adopt a biopsychosocial paradigm that inherently demands both of these cognitive functions [24,26]. Therefore, we propose that the baseline levels of executive function and cognitive flexibility will dictate the patient’s capacity to assimilate the educational intervention.

In the context of current literature, our protocol directly addresses several gaps identified in recent research. While recent meta-analyses and trials confirm that combining PNE with exercise yields significant clinical benefits in diverse populations, including older adults [28,36,37], most existing studies are limited by short intervention periods and a lack of long-term follow-ups. Furthermore, there is considerable heterogeneity regarding the optimal educational dosage. A recent dose–response meta-analysis established a threshold of 150 to 200 min of PNE to achieve clinically meaningful changes in pain and disability [42]. Our 8-week intensive phase (240 min) is specifically designed to safely exceed this threshold. However, considering the deep-rooted nature of maladaptive biomedical beliefs in older adults, short-term interventions might be insufficient to sustain behavioral changes. By introducing the 32-week maintenance arm, this trial expands upon current publications to explore whether sustained engagement and periodic reinforcement are required to consolidate pain reconceptualization and maintain active coping strategies over time.

From a methodological perspective, the design ensures that all participants in the three study arms benefit from a structured physical training program of the same duration and intensity. This guarantees an equivalent level of exposure to exercise, which will allow a rigorous and isolated evaluation of the true efficacy of PNE and its long-term dose–response effect. The study presents limitations inherent to non-pharmacological clinical trials, primarily the impossibility of applying double blinding (neither the participants nor the physiotherapist delivering the education can be blinded). However, this bias will be mitigated through a single-blind design, in which the evaluators in charge of data collection and the researchers performing the statistical analysis will be completely unaware of the group allocation.

## 6. Conclusions

This study protocol outlines a rigorous randomized controlled trial designed to address a critical gap in the management of geriatric chronic pain by evaluating the long-term dose–response effect of PNE combined with multicomponent exercise. By prioritizing active coping strategies over traditional passive treatments, this trial provides an innovative approach specifically tailored to the neurocognitive and physical needs of older adults. If our hypotheses are confirmed, the findings will generate high-quality evidence to determine the optimal educational dosage required for sustained pain relief. Ultimately, this will support the integration of combined active interventions into community settings, representing a safe and low-cost alternative centered on patient empowerment, promoting active aging, and reducing the burden of chronic pain in our communities.

## 7. Trial Registration

This trial protocol was prospectively registered at ClinicalTrials.gov (National Library of Medicine, USA) under the identifier: NCT07287501.

## Figures and Tables

**Table 1:** Description of the intervention protocols for the three study groups.

Feature	Control Group	Intervention Group 1 (IG1)	Intervention Group 2 (IG2)
Intervention Type	Multicomponent Exercise	Exercise + PNE (Short term)	Exercise + PNE (Long term)
Total Duration	32 weeks	32 weeks	32 weeks
Exercise Protocol	Frequency: 3×/week Duration: 50 min/session Content: Aerobic, Strength, Balance, Flexibility.	Frequency: 3×/week Duration: 50 min/session Content: Aerobic, Strength, Balance, Flexibility.	Frequency: 3×/week Duration: 50 min/session Content: Aerobic, Strength, Balance, Flexibility.
PNE Protocol	None	Duration: 8 weeks Frequency: 2×/week Total Dose: 240 min (16 sessions)	Duration: 32 weeks Phase 1: 8 weeks (2×/week) Phase 2: Weeks 9–32 (1×/2 weeks) Total Dose: 360 min (24 sessions)

**Table 2:** Structure, content, and objectives of the multicomponent exercise sessions.

Phase	Duration	Content	Objectives and Exercises
Warm-up	15 min	Active static and dynamic mobility	Objective: To prepare the musculoskeletal and cardiovascular systems. Exercises: Joint mobility and low-intensity movements during gait.
Main Phase	30–35 min	Aerobic, strength, and balance/coordination	Objective: To improve cardiovascular endurance, muscle power, and prevent falls. Exercises: Walking/marching, upper/lower limb resistance training, and dual-task balance challenges with cognitive stimuli (e.g., numbers, colors).
Cool-down	10 min	Stretching and breathing	Objective: To promote physical recovery and relaxation. Exercises: Static stretching of major muscle groups and guided diaphragmatic breathing.

**Table 3:** Content of the Pain Neuroscience Education (PNE) sessions.

Phase	Group	Session	Content and Pedagogical Objective
Phase 1: Intensive Educational Program (Weeks 1–8)	IG1 & IG2	Session 1	Presentation of the intervention, study objectives, and individual expectations. To establish a therapeutic alliance and align expectations.
Phase 1	IG1 & IG2	Session 2	Pain, perception, and context: Subjectivity of pain and the influence of environmental factors. To understand that pain is a subjective experience modulated by context.
Phase 1	IG1 & IG2	Session 3	Neurotag and sensory homunculus: How past memories and experiences affect pain. To recognize how past experiences and brain representation influence pain.
Phase 1	IG1 & IG2	Session 4	Acute pain vs. long-lasting pain: Protective function versus loss of protection (faulty alarm metaphor). To differentiate between the protective function of acute pain and the “faulty alarm” of chronic pain.
Phase 1	IG1 & IG2	Session 5	Peripheral and central sensitization: Understanding hypersensitivity and pain without a justified cause. To understand nervous system hypersensitivity without linking it to tissue damage.
Phase 1	IG1 & IG2	Session 6	Difference between damage and pain: Having pain does not necessarily mean there is a tissue injury. To decouple the concept of pain from structural injury.
Phase 1	IG1 & IG2	Session 7	Delivery of educational leaflet 1, knowledge verification questionnaire, and review of doubts. To consolidate theoretical knowledge and resolve doubts.
Phase 1	IG1 & IG2	Session 8	Introduction to active pain coping: Brainstorming tools to improve long-lasting pain. To transition from passive to active self-management tools.
Phase 1	IG1 & IG2	Session 9	Impact of chronic pain in older adults: Prevalence and the biopsychosocial complexity of pain. To validate the patient’s experience and introduce the biopsychosocial model.
Phase 1	IG1 & IG2	Session 10	Options for improvement: Limitations of imaging tests and drugs; importance of healthy daily habits (sleep, diet, social relations). To emphasize the role of lifestyle habits over passive medical interventions.
Phase 1	IG1 & IG2	Session 11	Exercise in chronic pain: Safety guidelines, postural hygiene, and graded exposure to movement. To reduce fear of movement and promote safe physical activity.
Phase 1	IG1 & IG2	Session 12	Attention and efference copy: Influence of attention on pain perception and how the brain automatizes pain. To explain how cognitive focus and automatization perpetuate the pain experience.
Phase 1	IG1 & IG2	Session 13	Neuroplasticity and relapses: The brain’s capacity to change and normalize worse moments during recovery. To foster hope through brain adaptability and normalize clinical setbacks.
Phase 1	IG1 & IG2	Session 14	Delivery of educational leaflet 2, knowledge verification questionnaire, and review of doubts. To consolidate theoretical knowledge and resolve doubts.
Phase 1	IG1 & IG2	Session 15	Resources and available social support: Community programs to improve quality of life and well-being. To integrate the patient into available community health resources.
Phase 1	IG1 & IG2	Session 16	General summary of the key concepts from the previous sessions, Q&A, and motivational closure. To empower the patient for the maintenance phase or independent management.
Phase 2: Maintenance and Behavioral Change (Weeks 9–32)	IG2	Session 17	General review of main concepts: Subjectivity, damage vs. pain, neuroplasticity, and the biopsychosocial approach. To refresh key pain reconceptualization concepts.
Phase 2	IG2	Session 18	Explanatory video on chronic pain and group discussion. To reinforce learning through multimedia and peer discussion.
Phase 2	IG2	Session 19	Confronting erroneous beliefs: Dynamic myths and realities using a color-coded card game. To actively confront and correct remaining erroneous beliefs.
Phase 2	IG2	Session 20	Problem-based learning: Clinical case study of an older woman with chronic pain to find practical solutions. To apply theoretical knowledge to solve real-life scenarios.
Phase 2	IG2	Session 21	Guided debate with practical situations: “What would I do?” dynamic to promote self-efficacy and active decision-making. To improve decision-making and self-efficacy in daily challenges.
Phase 2	IG2	Session 22	Role-playing: Simulating patient-professional conversations to practice empathetic and constructive responses. To practice assertive communication and empathetic understanding.
Phase 2	IG2	Session 23	Guidelines for a healthy diet and cardiac coherence technique (mindful breathing for anxiety reduction). To provide practical somatic tools for anxiety and inflammation reduction.
Phase 2	IG2	Session 24	Guided self-reflection: “What have I changed?”, assessing progress, and closing the program. To assess individual behavioral progress and close the program.

## Data Availability

Not applicable. As this is a study protocol, no new data were created or analyzed in this study. Data sharing is not applicable to this article.

## Abbreviations

The following abbreviations are used in this manuscript:

PNE	Pain Neuroscience Education
RCT	Randomized controlled trial
CONSORT	Consolidated Standards of Reporting Trials
GC	Control Group (CG)
IG1	Intervention Group 1
IG2	Intervention Group 2
PReGe	Programa de Revitalización Geriátrica
NRS	Numeric Rating Scale
TSK-11	Tampa Scale for Kinesiophobia
PCS	Pain Catastrophizing Scale
SPPB	Short Physical Performance Battery
TUG	Timed Up & Go
TMT	Trail Making Test
MCID	Minimal Clinically Important Difference
ANOVA	Analysis of Variance
REML	restricted maximum likelihood
